# Synthesis, Leishmanicidal Activity and Theoretical Evaluations of a Series of Substituted bis-2-Hydroxy-1,4-Naphthoquinones

**DOI:** 10.3390/molecules190915180

**Published:** 2014-09-22

**Authors:** Morgana V. de Araújo, Patricia S. O. de Souza, Aline C. de Queiroz, Carolina B. B. da Matta, Anderson Brandão Leite, Amanda Evelyn da Silva, José A. A. de França, Tania M. S. Silva, Celso A. Camara, Magna S. Alexandre-Moreira

**Affiliations:** 1 Laboratory of Pharmacology and Immunity, Institute of Biological Sciences and Health, Federal University of Alagoas, Maceió, AL 57020-720, Brazil; E-Mails: morgana_vital@hotmail.com (M.V.A.); allycq_farmacia@hotmail.com (A.C.Q.); carolina_damatta@hotmail.com (C.B.B.M.); bioufal@hotmail.com (A.B.L.); Amanda.evelyn13@hotmail.com (A.E.S.); 2 Laboratory of Bioactive Compounds Synthesis, Molecular Sciences Department, Federal Rural University of Pernambuco, Recife, PE 52171-900, Brazil; E-Mails: patti.soliveira@gmail.com (P.S.O.S.); adoniass@gmail.com (J.A.A.F.); sarmentosilva@gmail.com (T.M.S.S.); ccelso@gmail.com (C.A.C.)

**Keywords:** naphthoquinones, knoevenagel, bis-2-hydroxy-1,4-naphthoquinones, antileishmanial activity, *Leishmania braziliensis*, *Leishmania amazonensis*

## Abstract

A series of eight substituted bis-2-hydroxy-1,4-naphthoquinone derivatives was synthesized through lawsone condensation with various aromatic and aliphatic aldehydes under mild acidic conditions. The title compounds were evaluated for antileishmanial activity *in vitro* against *Leishmania amazonensis* and *Leishmania braziliensis* promastigotes; six compounds showed good activity without significant toxic effects. The compound with the highest activity was used for an *in vivo* assay with *Leishmania amazonensis*.

## 1. Introduction

Quinones are a well-known class of compounds with a broad natural distribution; they exhibit a diverse spectra of biological activities, including antitumor [1,2], molluscicidal [3], leishmanicidal [4,5], bactericidal, fungicidal [6,7] and trypanocidal functions [8], and they act as inhibitors of the reverse transcriptase enzyme of HIV-1 [9] and human topoisomerase II [10]. The conjugated 1,4-dicarbonyl or 1,2-dicarbonyl moiety in the most well-known *para*- and *ortho*-quinone molecular structures confers specific properties and a reactivity that facilitates participation in redox processes, which are likely related to these substances’ mechanisms of action [11,12].

Studies that have considered natural compounds with leishmanicidal activity have highlighted diospyrin, which is a bis-naphthoquinone isolated from *Diospyros montana* (Ebenaceae) bark; plumbagin isolated from *Plumbago* species (Plumbaginaceae); and lapachol, which is a prenylated hydroxynaphthoquinone isolated from *Tecoma* species (Bignoniaceae). These compounds represent a class of quinones with a mechanism of action that involves generating oxygen free radicals, which affect the parasites’ defense mechanism and renders the parasites defenseless [13].

Dimeric structures with two 2-hydroxy-1,4-naphthoquinone groups have been studied primarily as intermediates in benzoxanthene derivative synthesis [14,15,16]; however, a few studies have investigated the biological activities of these compounds. Mazumder and co-workers [17] studied the inhibitory activity of the HIV integrase enzyme using a series of bis-2-hydroxy-1,4-naphthoquinones and reported good results.

In this study, a series of bis-2-hydroxy-1,4-naphthoquinones (bis-lawsones) were synthesized using a simple and quick method. These compounds were evaluated *in vitro* for antileishmanial activity against *Leishmania amazonensis* and *Leishmania braziliensis* promastigotes. The most active compound was used for an *in vivo* assay with *Leishmania amazonensis*.

## 2. Results and Discussion

**Scheme 1:**
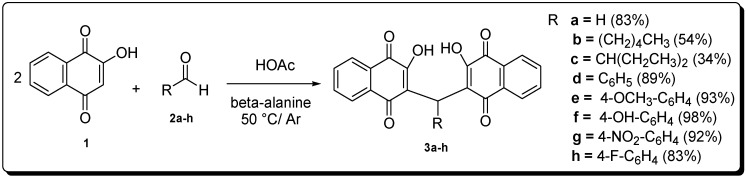
Synthesis of compounds **1**–**3h**.

Compounds **3a**–**h** (Scheme 1) were obtained in good yields from a condensation reaction between lawsone with the corresponding aldehyde using β-alanine and acetic acid catalysis in an inert atmosphere at 50 °C. The compounds **3b** and **3c** obtained from the aliphatic aldehydes **2b** and **2c** showed lower yields due to the formation of the corresponding alkene byproducts through a condensation side reaction that yielded the corresponding 2-alkenyl derivative [15,16].

Primarily, the bis-lawsone analogs’ cytotoxicity was determined using the MTT method [18] and J774 cell line. The host cells were treated with bis-lawsone analogs and compared with the vehicle (DMSO). Table 1 shows the results for the bis-lawsone analogs and pentamidine (reference drug) experiments. The compounds **3c** and pentamidine showed the same deleterious activity to the host cell, as evidenced by the MTT assay, which presented the maximum cytotoxicities 71.7% ± 3.8% and 78.0% ± 3.8% as well as LC_50_ values at 67.4 ± 2.1 and 73.0 ± 6.0 µM, respectively. After 48 h of incubation, the other compounds did not affect the J774 cell line viability at 100 µM.

**Table 1:** Determination of the cytotoxicity of bis-lawsone analogs **3a**–**h** against macrophages (MTT assay).

Compounds	Chemical Structure (R=) 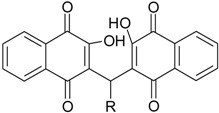	LC_50_(µM) ^a^	Maximum Cytotoxicity (%) ^b^
Pentamidine	-	73.0 ± 6.0	78.0 ± 3.8 **
**3a**	H	>100	NT
**3b**	n-Pentyl	>100	NT
**3c**	CH(Et)_2_	67.4 ± 2.1	71.7 ± 3.8 **
**3d**	-Ph	>100	NT
**3e**	-C_6_H_4_(4-OMe)	>100	NT
**3f**	-C_6_H_4_(4-OH)	>100	NT
**3g**	-C_6_H_4_(4-NO_2_)	>100	NT
**3h**	-C_6_H_4_(4-F)	>100	NT

^a^ Lethal Concentration 50 (LC_50_) calculated by concentration-response curves toxic, using as maximal concentration 100 μM; ^b^ Mean ± standard error of the mean maximum cytotoxicity in triplicates of a representative experiment; The values of maximum effect were considered significant when ** *p* < 0.01 compared to the DMSO group; NT: substance presents no significant lethal activity to cell until the concentration of 100 M in compared to DMSO group.

**Figure 1:**
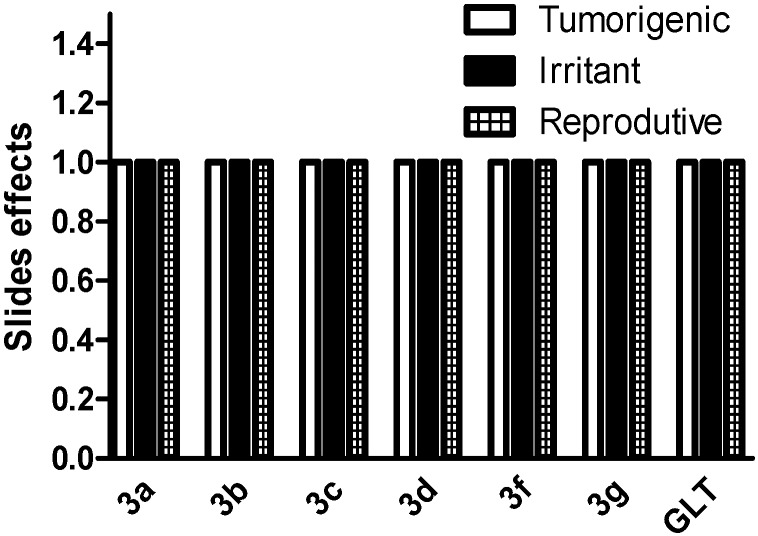
*In silico* toxicity risk (tumorigenic, irritant and reproductive effects) for lawsone dimers. Theoretical toxicity risks calculated using the Osiris program. The toxicity profile scale for the side effects included low (1), medium (0.8) and high (0.6).

**Figure 2:**
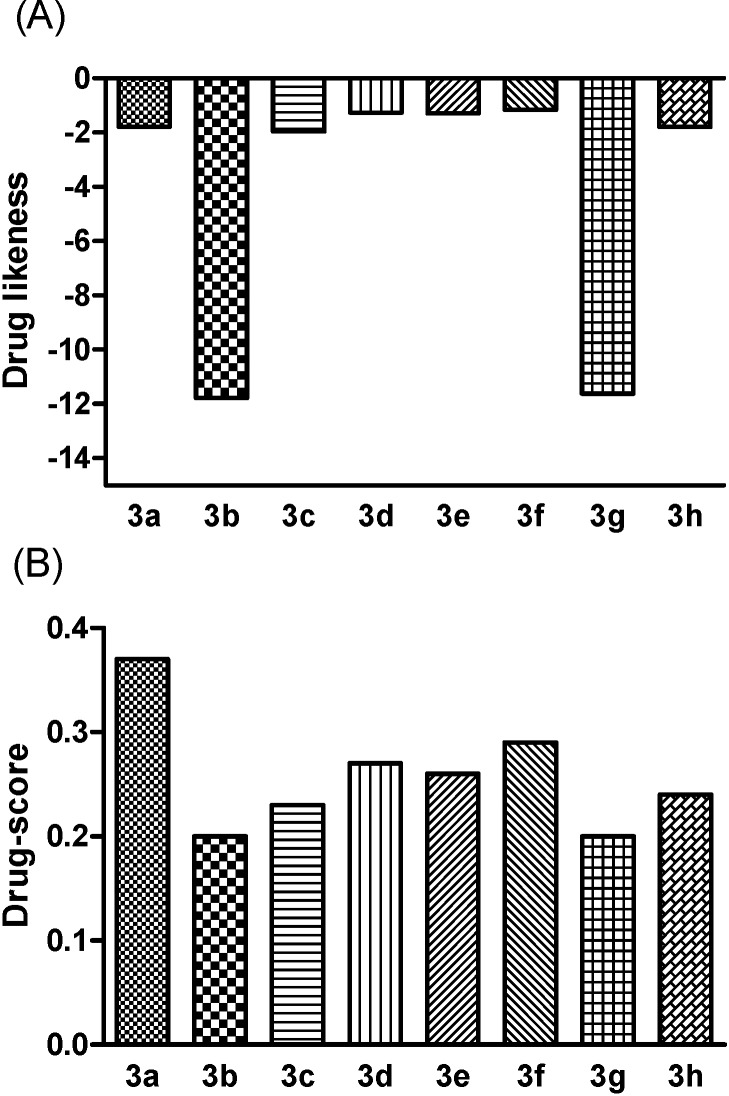
*In silico* comparison of the drug-like profile (drug-likeness (**A**) and drug score values (**B**)) for the bis-lawsone analogs and glucantime (GLT). These parameters were calculated using the Osiris program as described in the Experimental Section.

Theoretical toxicity analysis was performed using the OSIRIS program [18] to analyze their overall drug score and drug-likeness potential as well as toxicity risks (mutagenic, irritant, tumorigenic and reproductive effects) [19]. Comparing the compounds **3a**–**h** to the available drugs currently used for the treatment of leishmaniasis, the results show that the bis-lawsone derivatives had no toxicity effects (Figure 1). Except **3c**, these results are consistent with the MTT assay results (Table 1). Notably, the toxicity predicted herein is neither a fully reliable toxicity prediction nor a guarantee that these compounds are completely free of a toxic effect, however, the data reinforce the promising profiles for these compounds, which were also detected *in vitro*, for further experimental investigation. Further, the bis-lawsone analogs’ drug-like profiles (drug likenesses and drug-score values) were determined using the OSIRIS program (Figure 2).

The OSIRIS program calculates the drug-likeness based on a list of about 5300 distinct substructure fragments created from 3300 traded drugs as well as 15,000 commercially available chemicals, yielding a complete list of all available fragments with associated drug-likeness. A positive value states that the molecule contains predominately fragments which are frequently present in commercial drugs, so the bis-lawsone analogs do not present these fragments. The drug score already combines drug-likeness, cLogP, logS, molecular weight and toxicity risks in one handy value that may be used to judge a compound’s overall potential to qualify as a drug. Thus, it was found that **3a** showed the highest drug score among all synthesized analogs.

To establish the leishmanicidal profile, the bis-lawsone analogs were evaluated *in vitro* against the forms of *L. amazonensis* and *L. braziliensis*. Pentamidine was used as reference drug in the *in vitro* tests to evaluate leishmanicidal activity because glucantime (meglumine antimoniate) is not active against promastigote forms. As a parameter for antileishmanial activity, the maximum effect and IC_50_ value (*i.e.*, the sample concentration that reduces survival/viability of the parasites by 50%) were used (Table 2). As shown, the compounds **3a** and **3c** were highly active against both *Leishmania* species, presenting the effects 72.8% ± 1.0% and 75.3% ± 1.3% against *L. amazonensis* and killed promastigotes of *L. braziliensis* in the proportion of 88.4% ± 0.9% and 90.4% ± 0.7%, respectively. In addition, **3b**, **3e**, **3f**, **3g** and **3h** exhibited high antileishmanial activities against *L. braziliensis* promastigotes with the maximum effects 93.0% ± 0.1%, 61.6% ± 7.5%, 91.7% ± 0.3%, 91.7% ± 1.3% and 88.7% ± 0.3%, respectively. Moreover, the bis-lawsone analogs **3a** (IC_50_ value 0.9 ± 0.08 µM), **3b** (IC_50_ value 5.2 ± 0.1 µM), **3e** (IC_50_ value 0.9 ± 0.04 µM) and **3h** (IC_50_ value 0.8 ± 0.03 µM) were as potent as pentamidine (with the efficacy 91.0% ± 0.1% and IC_50_ value 0.8 ± 0.06 µM) for this *Leishmania* species. In contrast, the derivative **3d** did not present activity against promastigote forms of *L. braziliensis* until 100 µM; however, it showed a considerable effect (85.0% ± 3.8%) and great potency (IC_50_ value 0.3 ± 0.1 µM) against *L. amazonensis*. The compounds **3a** and **3e** were more selective against *L. braziliensis*, presenting IC_50_ values to this specie 10 times lower than IC_50_ value to *L. amazonensis*. On the contrary, compounds **3c** and **3d** were more selective to *L. amazonensis* in comparation to *L. amazonensis*. On the other hand, the other compounds showed similar activities against both species of *Leishmania* tested.

**Table 2:** Leishmanicidal effect of bis-lawsone analogs **3a**–**h** against the growth of promastigotes of *L. amazonensis* and *L. braziliensis*.

Compounds	Chemical Structure (R=) 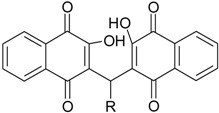	*L. amazonensis*		*L. braziliensis*	
		IC_50_ (µM) ^a^	Maximum Effect (%) ^b^	IC_50_ (µM) ^a^	MaximumEffect (%) ^b^
Pentamidine	-	2.3 ± 0.8	85.4 ± 0.4 **	0.8 ± 0.06	91.0 ± 0.1 **
3a	H	**71.0 ± 1.1**	72.8 ± 1.0 **	**0.9 ± 0.08**	88.4 ± 0.9 **
3b	*n*-Pentyl	5.2 ± 0.1	57.2 ± 3.8 **	**5.2 ± 0.1**	93.0 ± 0.1 **
3c	CH(Et)_2_	**0.4 ± 0.1**	75.3 ± 1.3 **	**34.7 ± 4.3**	90.4 ± 0.7 **
3d	Ph	**0.3 ± 0.1**	85.0 ± 3.8 **	>100	NT
3e	(4-OMe)Ph	>100	44.6 ± 2.4 **	**0.9 ± 0.04**	61.6 ± 7.5 **
3f	(4-OH)Ph	68.7 ± 15.1	55.4 ± 4.7 **	**38.7 ± 2.0**	91.7 ± 0.3 **
3g	(4-NO_2_)Ph	7.7 ± 1.2	55.6 ± 3.2 **	**2.8 ± 0.1**	91.7 ± 1.3 **
3h	(4-F)Ph	0.6 ± 0.2	51.0 ± 5.5 **	**0.8 ± 0.03**	88.7 ± 0.3 **

^a^ Inhibitory Concentration 50 (IC_50_) was calculated by concentration-response curves toxic and expressed as mean ± standard error of the mean, using as maximal concentration 100 μM; ^b^ Maximum Effect (ME) is expressed as mean ± standard error of maximum toxicity average of triplicates of a representative experiment; The values of maximum effect were considered significant when ** *p* < 0.01 compared to the 0.1% DMSO group; NT: substance presents no significant inhibitory activity for the parasite to the concentration of 100 M compared to DMSO group.

These results indicate a correlation between the leishmanicidal activity and the presence of an alkyl side-chain moiety with a hydrophobic character for the compounds in the series **3a**–**c**. The compounds with the non polar groups generally exhibit lower activities (Table 2); however, the exception to this trend is the compound **3d**, likely due to an oxidative metabolic pathway at the free phenyl ring 4-position. The capacity for further oxidation exhibited by the derivative **3f** with a free *p*-hydroxyl seems to support this assumption. Moreover, the electron-withdrawing substituents in the phenyl ring, as demonstrated for **3h**, seems more selective compared with a 4-methoxy donor (**3e**) in *L. amazonensis*; both show a similar profile in *L brasiliensis*.

Considering the *in vitro* results, the compound **3a** was also used to evaluate the *in vivo* leishmanicidal activity against *L. amazonensis*. Glucantime was used as control in the *in vivo* assay, as this reference drug is the first choice treatment for leishmaniasis and has a lower toxicity to mice, killing a lesser number of animals compared to pentamidine. Glucantime can also be administrated by intraperitoneal route, unlike pentamidine that is just administrated by intravenous route.

**Figure 3:**
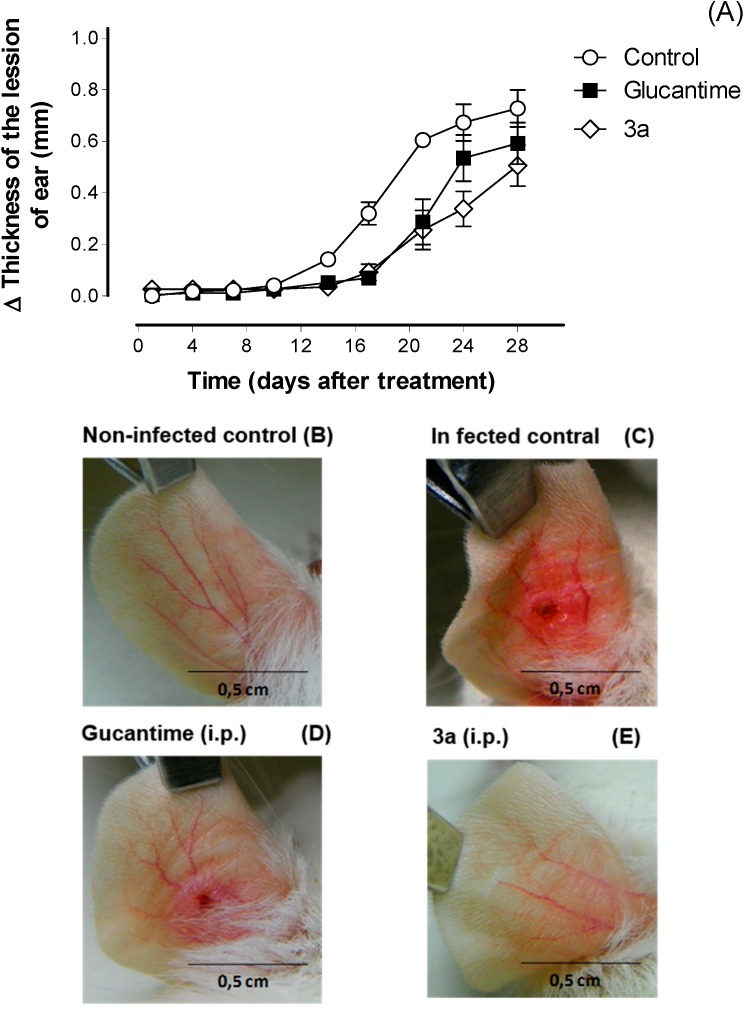
*In vivo* efficacy of **3a** and glucantime treatments (30 μmol/kg/day × 28 days, i.p.) (**A**) in BALB/c mice infected wituh *L. amazonensis*. The lesion sizes were monitored weekly (non-infected control (**B**), infected control (**C**), glucantime (**D**) and **3a** (**E**)). The values are the mean lesion sizes for five mice from each group, and the bars represent the standard error of the mean.

**Figure 4:**
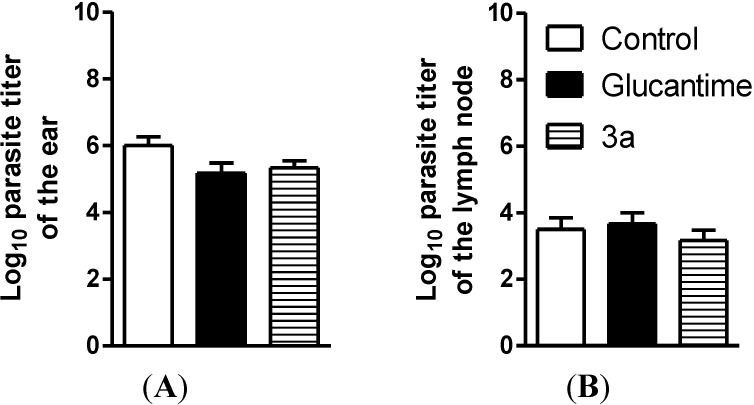
Parasite burden throughout the course of the **3a** and glucantime treatments (30 μmol/kg/dia × 28 days, i.p.) in BALB/c mice infected with *L. amazonensis*. (**A**) Log_10_ of the parasites load in the infected ear; (**B**) Log_10_ of the parasites load in the draining lymph node. The infected ear and draining lymph node parasite loads were determined using a quantitative limiting-dilution assay. Values are the mean parasites load for five mice from each group, and the bars represent the standard error of the mean.

**Figure 5:**
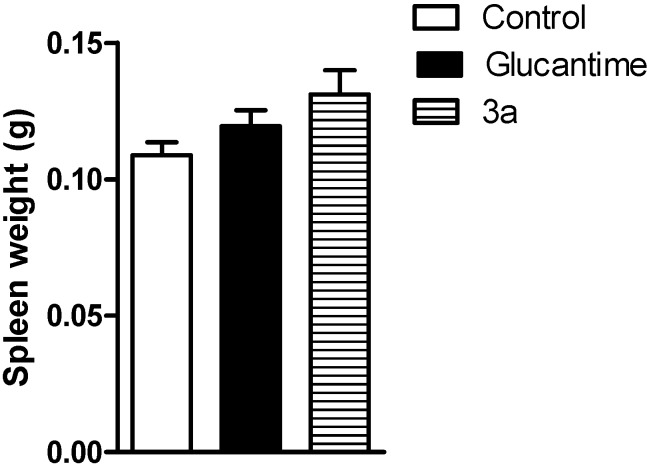
The *in vivo* effect from the **3a** and glucantime treatments (30 μmol/kg/day × 28 days, i.p.) on the spleen weights of the BALB/c mice infected with *L. amazonensis*. The spleen weight was determined on last day of treatment. The values are the mean lesion size for five mice from each group, and the bars represent the standard error of the mean.

**Figure 6:**
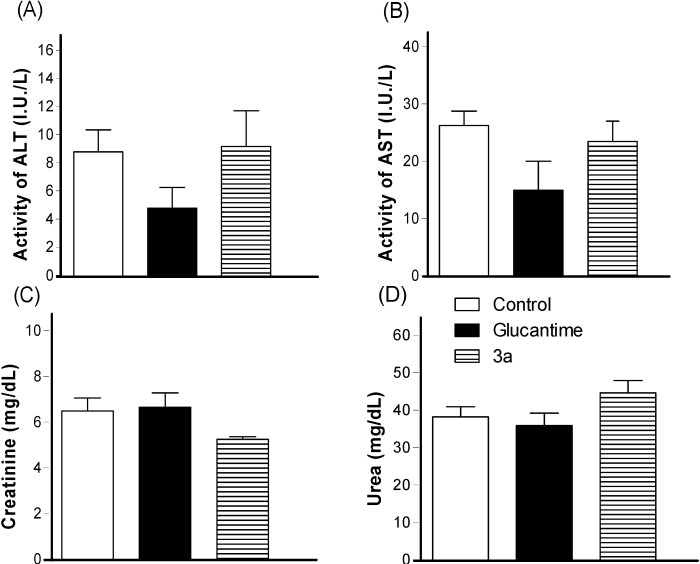
The *in vivo* effect from the **3a** and glucantime treatments (30 μmol/k/day × 28 days, i.p.) on the serum ALT (**A**), AST (**B**), creatinine (**C**) and urea (**D**) levels in BALB/c mice infected with *L. amazonensis*. The lesion sizes were monitored weekly. The values are the mean lesion size for five mice from each group, and the bars represent the standard error of the mean.

Intraperitoneal treatment with **3a** at 30 μmol/kg/day × 28 days decreased the lesion size for the infected ear on the third week after the treatment began (Figure 3); it did not decrease the parasite load in the infected ear and draining lymph node, similar to the glucantime treatment at same dose (Figure 4). The effect on the lesions obtained with **3a** treatment can be due to a possible immunomodulatory or anti-inflammatory activity of the analogue as well because there was a decrease in virulence of the parasite during treatment, although there isn’t reduction in parasite burden. In addition, *in vivo* treatment with **3a** neither induced a change in the spleen weight (Figure 5) nor altered alanine aminotransferase (ALT), aspartate aminotransferase (AST), creatinine (CREA) and urea (Figure 6) in the animals’ plasma.

Among the known biological activities of compounds with a quinone structure, these substances stand out for their antiprotozoal activity. For example, three synthesized naphthofuranquinone C-allyl lawsone derivatives were active against *Trypanosoma cruzi* trypomastigotes [8]. When used as therapeutic agents, quinonoidal ligand cytotoxic activity operates through various mechanisms, such as redox cycling, intercalation, inducing DNA strand breaks, arylation, alkylation via quinone methide formation and free radical generation [20].

Leishmaniasis is a public health issue and is among the five most prevalent parasitic diseases worldwide. According to the World Health Organization, anthropozoonosis leishmaniasis affects 12 million people with an annual incidence of approximately 2 million new cases, and most occur in undeveloped countries such as Brazil. The standard leishmaniasis treatment includes antimonials, amphotericin B and pentamidine, but these compounds are often associated with serious side effects. [21] Discovery and development of new therapeutic agents is a priority due to the increasing prevalence of drug resistance in *Leishmania*, toxicity towards currently used drugs and the lack of an effective prophylactic vaccine against the disease [22].

Naphthoquinone antileishmanial activity has also been observed. Lapachol, isolapachol and dihydrolapachol present significant activity; isolapachol acetate was the most active against promastigotes, with IC_50_ values of 1.6 μg/mL and 3.4 μg/ml for *L. amazonensis* and *L. braziliensis*, respectively [23]. A series of monomeric and dimeric naphthoquinones with potential for treating *Leishmania* infections was identified *in vitro* against extracellular *L. donovani*, *L. infantum*, *L. enriettii* and *L. major* promastigotes. Several naphthoquinones were actives at microgram concentrations (EC_50_ 0.9–17.0 μg/mL). When tested against a panel of human cancer cell lines and murine bone marrow culture-derived macrophages as mammalian host cell controls, compounds with anti-leishmanial activity showed moderate (EC_50_ 25.0 μg/mL) to pronounced (EC_50_ 10.0 μg/mL) toxic effects [24]. Our study also verified that most of the naphthoquinones tested noticeably inhibited extracellular parasite growth (IC_50_ 0.8–38.7 μM) of *L. braziliensis*.

While the mechanism of action of the naphthoquinones to kill these parasites is a matter of debate, biochemical experiments provide initial insights. For example, it known that at ovaquone interacts with cytochrome Bl-c in *Plasmodium* spp. as well as ubiquinone is can be the potential target of analogs of the coenzyme Q, such as naphthoquinones [25,26]. This theory supports the hypothesis by Croft and co-workers [27,28] wherein killing *Leishmania* through a series of previously tested monomeric naphthoquinones involves generating free radicals when the drug interacts with the respiratory chain. In addition, the bis-lawsone analogs exhibit leishmanicidal activity against *L. donovani* promastigotes (with the IC_50_ values ranging from 2 to 14 µM) and inhibit leishmanial DNA topoisomerase-I [29]. Moreover, Plyta *et al.* [30] showed that 1,4-naphthoquinones bearing at least one phenolic hydroxyl group are potent topoisomerase enzyme inhibitors. Therefore, we will continue this study to evaluate the inhibitory effects of theses analogs on topoisomerase of *Leishmania* as well as other validated chemotherapeutic target.

## 3. Experimental Section

### 3.1. General Information

All reagents and solvents were obtained from commercial suppliers and used without further purification. The reaction progress was monitored using thin layer chromatography on silica gel TLC aluminum sheets. The melting points were determined using a Kofler hot stage apparatus and are uncorrected. FTIR spectra were obtained in a BOMEM MB-Series 100 spectrophotometer or a Bruker IRS66 using KBr discs. The NMR spectra were recorded in a Varian Unity Plus 300 or Varian UNMRS 400 instrument. Elemental analyses were performed using a CE EA1110 CHNS-O analyzer.

### 3.2. Chemistry

#### General Procedure for the Synthesis of Compounds **3a**–**h**

A solution of lawsone (**1**, 174 mg, 1 mmol), the corresponding aldehyde **2a**–**h** (0.5 mmol), β-alanine (15 mg) and 2.5 mL of glacial acetic acid was stirred at 50 °C in argon atmosphere and reflux system for periods of 90 min at 5 h according to aldehyde used (the progress of reaction was monitored by TLC). The isolation of the compounds was done by adding crushed ice followed (except for compounds **3b** and **3c**) by vacuum filtration of the formed precipitates. The solids were washed with distilled water and dried at room temperature, and the compounds were pure enough as inspected by TLC and spectroscopic methods [16]. For compounds **3b** and **3c** after adding ice the mixture was extracted with ethyl acetate, dried with anhydrous sodium sulfate and the solvents removed under reduced pressure, followed by subsequent purification in chromatography column with silica gel as stationary phase and methylene chloride as mobile phase.

*3,3'-(Methylene)-bis[2-hydroxy-1,4-naphthalenedione]* (**3a**). Yield: 83% yellow solid. mp: 229–232 °C dec. (229 °C) [31]. IR (KBr) ν_max_/cm^−1^: 3452, 3070, 1678, 1610, 1573, 1458, 1350, 1323, 1265, 1215, 975, 937, 771, 736, 466; ^1^H-NMR (DMSO-*d*_6_, 300 MHz) δ 3.74 (s, 2H), 7.78 (dt, 2H, *J* 7.5/1.8 Hz), 7.83 (dt, 2H, *J* 7.5/1.5 Hz), 7.97 (m, 4H); ^13^C-NMR(DMSO-*d*_6_, 75.4 MHz) δ 17.9, 122.0, 125.7, 125.9, 129.9, 132.0, 133.2, 134.6, 155.1, 180.8, 183.6. Anal. Calcd. C_21_H_12_O_6_: C, 70.00; H, 3.36. Found: C, 69.03; H, 3.82.

*3,3'-(Hexylidene)-bis[2-hydroxy-1,4-naphthalenedione]* (**3b**). Yield: 54%, orange solid. mp: 205–208 °C dec. (213 °C) [32]. IR (KBr) ν_max_/cm^−1^: 3433, 2928, 2855, 1667, 1628, 1585, 1566, 1458, 1366, 1281, 953, 737; ^1^H-NMR (DMSO-*d*_6_, 300 MHz) δ 1.32 (t, 3H, *J* 6.6Hz), 1.72 (m, 6H), 3.05 (m, 2H), 5.83 (t, 1H, *J* 8.1 Hz), 8.17 (dt, 2H, *J* 7.5/1.5 Hz), 8.27 (dt, 2H, *J* 7.8/1.5 Hz), 8.38 (dd, 2H, *J* 7.5/1.5 Hz), 8.46 (dd, 2H, *J* 7.8/1.5 Hz); ^13^C-NMR (DMSO-*d*_6_, 75.4 MHz) δ 14.0, 22.1, 27.6, 28.8, 29.1, 31.4, 123.7, 125.0, 125.7, 130.6, 132.0, 133.1, 133.9, 162.6, 182.9, 183.6. Anal. Calcd. C_26_H_22_O_6._(2H_2_O): C, 66.95; H, 5.63. Found: C, 68.96; H, 5.74.

*3,3'-(2-Ethylbutylidene)-bis[2-hydroxy-1,4-naphthalenedione]* (**3c**). Yield: 34%, orange solid. mp 210–211 °C (169–170 °C) [33] IR (KBr) ν_max_/cm^−1^: 3438, 2962, 2930, 1649, 1597, 1461, 1364, 1283, 729; ^1^H-NMR (DMSO-*d*_6_, 400 MHz) δ 0.71 (t, 6H, *J* 7.2 Hz), 1.16 (m, 2H),1.31 (m, 2H), 2.8 (m, 1H), 5.20 (d, 1H, *J* 12.4 Hz), 7.63 (t, 2H, *J* 7.2 Hz), 7.72 (t, 2H, *J* 7.6 Hz), 7.84 ( d, 2H, *J* 7.6 Hz), 7.90 (d, 2H, *J* 7.6 Hz); ^13^C-NMR (DMSO-*d*_6_, 75.4 MHz) δ 10.0, 22.0, 33.7, 35.6, 123.4, 125.2, 125.9, 130.3, 132.4, 132.8, 134.2, 160.6, 182.7, 183.5. Elemental Anal. Calcd. C_26_H_22_O_6_: C, 72.55; H, 5.15. Found: C, 67.50; H, 5.40.

*3,3'-(Phenylmethylene)-bis[2-hydroxy-1,4-naphthalenedione]* (**3d**). Yield: 89%, yellow solid. mp 215–217 °C (202–204 °C) [16]. IR (KBr) ν_max_/cm^−1^: 3450, 1674, 1597, 1570, 1361, 1284, 1222, 1111, 1056, 729; ^1^H-NMR (DMSO-*d*_6_, 300 MHz) δ 6.69 (s, 1H), 7.11 (m, 5H), 7.67 (dt, 2H, *J* 7.5/1.2Hz), 7.76 (dt, 2H, *J* 7.5/1.2 Hz), 7.88 (dd, 2H, *J* 7.5/1.2 Hz), 7.97 (dd, 2H, *J* 7.5/ 1.2 Hz); ^13^C-NMR (DMSO-*d*_6_, 100 MHz) δ 37.7, 123.1, 125.4, 125.6, 126.0, 127.6, 128.2, 129.9, 132.2, 133.1, 134.7, 140.8, 156.3, 181.2, 183.5. Anal. Calcd. for C_27_H_16_O_6_: C, 74.31; H, 3.70. Found: C, 74.16; H, 4.28.

*3,3'-(4-Methoxyphenylmethylene)-bis[2-hydroxy-1,4-naphthalenedione]* (**3e**). Yield: 93%, yellow solid. mp 222–223 °C (220–222 °C) [16].IR (KBr) ν_max_/cm^−1^: 3394, 1666, 1639, 1593, 1512, 1458, 1361, 1338, 1276, 1261, 1238, 1045, 1018, 721; ^1^H-NMR (DMSO-*d*_6_, 300 MHz) δ 3.69 (s, 3H), 5.94 (s, 1H), 6.75 (d, 2H, *J* 8.7 Hz), 7.14 (d, 2H, 8.7 Hz), 7.77 (dt, 2H, *J* 7.5/1.5 Hz), 7.82 (dt, 2H, *J* 7.5/1.5 Hz), 7.92 (dd, 2H, *J* 7.5/1.5 Hz), 7.98 (dd, 2H, *J* 7.5/1.5 Hz); ^13^C-NMR (DMSO-*d*_6_, 75.4 MHz) δ 37.3, 54.9, 113.1, 123.6, 125.6, 126.1, 129.4, 129.9, 132.2, 132.7, 133.2, 134.7, 155.9, 157.3, 181.3, 183.7. Anal. Calcd. C_28_H_18_O_7_: C, 72.10; H, 3.89. Found: C, 70.49; H, 3.98.

*3,3'-(4-Hydroxyphenylmethylene)-bis[2-hydroxy-1,4-naphthalenedione]* (**3f**). Yield: 98%, yellow solid. mp 175–176 °C (175–177 °C) [16]. IV (KBr) ν_max_/cm^−1^: 3352, 1647, 1593, 1512, 1458, 1365, 1276, 1045, 1010, 972, 902, 725; ^1^H-NMR (DMSO-*d*_6_, 300 MHz) δ 5.92 (s, 1H), 6.58 (d, 2H, *J* 8.7 Hz), 7.01 (d, 2H, *J* 8.7 Hz), 7.76, (dt, 2H, *J* 7.5/1.2Hz), 7.81 (dt, 2H *J* 7.5/1.8 Hz), 7,92 (dd, 2H, *J* 7.5/1.2 Hz), 7.97 (dd, 2H, *J* 7.5/1.8 Hz); ^13^C-NMR (DMSO-*d*_6_, 75.4 MHz) δ 37.2, 114.6, 123.8, 125.6, 126.8, 129.3, 129.8, 130.8, 132.2, 133.2, 134.7, 155.3, 155.9, 181.3, 183.9. Anal. Calcd. C_27_H_16_O_7_(2H_2_O): C, 66.40; H, 4.14. Found: C, 67.91; H, 4.38.

*3,3'-(4-Nitrophenylmethylene)-bis[2-hydroxy-1,4-naphthalenedione]* (**3g**). Yield: 92%, yellow solid. mp 143–146 °C. (177–179 °C) [16]. IR (KBr) ν_max_/cm^−1^: 3433, 2924, 1670, 1597, 1570, 1512, 1350, 1280, 111, 732; ^1^H-NMR (DMSO-*d*_6_, 300 MHz) δ 6.08 (s, 1H), 7.53 (d, 2H *J* 6.3 Hz), 7.78 (dt, 2H *J* 5.7/0.9 Hz), 7.83 (dt, 2H, *J* 5.7/0.9 Hz) 7.93 (dd, 2H, *J* 5.7/0.9 Hz), 8.00 (dd, 2H, *J* 5.7/0.9 Hz), 8.07 (d, 2H, *J* 6.6 Hz); ^13^C-NMR (DMSO-*d*_6_, 75.4 MHz) δ 37.6, 121.8, 122.8, 125.7, 126.1, 129.3, 130.0, 132.2, 133.2, 134.7, 145.5, 150.0, 156.9, 181.2, 183.3. Anal. Calcd. C_27_H_15_NO_8_: C, 67.36; H, 3.14; N, 2.91. Found: C, 65.79; H, 3.63; N, 3.63.

*3,3'-(4-Fluorophenylmethylene)-bis[2-hydroxy-1,4-naphthalenedione]* (**3h**). Yield: 83%, yellow solid. mp 202–204 °C (193–195 °C) [14]. IR (KBr) ν_max_/cm^−1^: 3414, 3348, 1666, 1625, 1593, 1508, 1458, 1365, 1342, 1276, 1230, 1161, 1041, 833, 725; ^1^H-NMR (DMSO-*d*_6_, 400 MHz) δ 6.00 (s, 1H), 7.00 (m, 2H), 7.28 (m, 2H), 7.77 (m, 4H), 7.91 (d, 2H, *J* 7.2 Hz), 7.97 (d, 2H, 6.4 Hz); ^13^C-NMR (DMSO-*d*_6_, 100 MHz) δ 37.7, 114.5, 123.5, 126.0, 126.5, 130.3, 132.6, 133.5, 135.1, 137.4, 156.5, 159.8, 162.2, 181.6, 184.0. Anal. Calcd. C_27_H_15_FO_6_: C, 71.37; H, 3.33. Found: C, 71.16; H, 3.44.

### 3.3. Biological Evaluation of Naphthoquinones

#### 3.3.1. Parasite Culture

*L. braziliensis* promastigotes (MHOM/BR/87/BA788) were obtained from Dra. Valéria de Matos Borges at the Gonçalo Moniz Research Center, Fiocruz_BA. *L. amazonensis* promastigotes (MHOM/BR/77/LTB0016) were obtained from Dr. Eduardo Caio Torres dos Santos at the Oswaldo Cruz Institute-Fiocruz_RJ. The parasites were maintained *in vitro* in Schneider’s medium supplemented with 10% FBS and 2% human urine at 27 °C in BOD incubator.

#### 3.3.2. J774.A1 Murine Macrophage Culture

The adherent-phenotype macrophage line was cultured in Dulbecco’s Modified Eagle’s medium (DMEM, Sigma, Dublin, Ireland) supplemented with 10% FBS at 37 °C, 95% humidity and 5% CO_2_.

#### 3.3.3. Cytotoxicity against Host Cells

To evaluate the cytotoxicity activity against the J774 cell line, the host cells were plated in 96-well vessels at 2 × 10^5^ cells per well in a complete culture medium with 10% FBS at 37 °C. After 1 h, the wells were washed with HBSS to remove non-adherent cells, leaving approximately 1 × 10^5^ adherent macrophages. The cells were cultured in DMEM complete medium supplemented with 10% FBS. The compounds and pentamidine were added at serial concentrations (0.1–100 µM). The cells were also cultured in media free from compounds, a vehicle (basal growth control) or media with DMSO 0.1% (vehicle control). The positive control (dead cells) was obtained through cellular lyses with 1% of Triton X-100 in DMEM complete medium. After 48 h, the cytotoxicity was evaluated using the MTT assay [34]. The data obtained from the experiments were expressed as the mean ± S.E.M., and the significant differences between the treated and vehicle groups were evaluated using ANOVA and Dunnett *post-hoc* tests.

#### 3.3.4. *In Vitro* Activity against *L. braziliensis* and *L. amazonensis*

The parasites were maintained *in vitro* in Schneider’s medium supplemented with 10% FBS and 2% human urine. Novel bis-lawsone analog stock solutions and pentamidine (reference leishmanicidal drug) were prepared in DMSO immediately before use. The cytotoxicities of the bis-lawsone analogs and pentamidine against the promastigotes were determined. Stationary phase *L. braziliensis* and *L. amazonensis* promastigotes were plated in 96-well vessels (Nunc, Roskilde, Denmark) at 1 × 10^5^ cells per well in Schneider’s medium supplemented with 10% FBS and 2% human urine. Each compound solution was added at increasing concentrations (0.1–100 mg to the extract and its phases or 0.1–100 µΜ to the isolates and pentamidine). The cells were also cultured in a medium free of compounds, a vehicle (basal growth control) or with DMSO 0.1% (vehicle control). After 48 h, the extracellular load for *L. braziliensis* and *L. amazonensis* promastigotes was estimated by counting the promastigotes in Schneider’s medium using a CELM automatic cell counter (model CC530, Barueri, Brazil) [35]. The data obtained from experiments were expressed as the mean ± S.E.M., and the significant differences between the treated and vehicle groups were evaluated using ANOVA and Dunnett *post-hoc* tests.

#### 3.3.5. *In Vivo* Activity against *Leishmania amazonensis*

This study was approved (protocol no. 2013.02) by the Ethics Committee for Animal Experimentation of the Federal University of Alagoas (Brazil). All animals received humane care in compliance with the “Principles of laboratory animal care” formulated by the National Society for Medical Research and the “Guide for the care and use of laboratory animals” prepared by the National Academy of Sciences (Washington, DC, USA). Next, 1 × 10^5^ stationary promastigotes (5 days of culture in Schneider’s medium) of *L. amazonensis* (MHOM/BR/77/LTB0016) were subcutaneously inoculated into the right ear dermis of 6-week-old female BALB/c mice weighing *ca.* 20 g. and were later intraperitoneally treated with 3a or glucantime at 30 μmol/kg × 28 days. The lesion size was measured using a paquimeter [36]. The parasite loads of infected ears and draining lymph nodes were determined using a quantitative limiting-dilution assay [37]. Complex toxicity was also evaluated through biochemistry dosages in plasma. The experimental data were expressed as the mean ± S.E.M., and the significant differences between the treated and vehicle groups were evaluated using ANOVA and Dunnett *post-hoc* tests.

#### 3.3.6. *In Silico* Screening

Bis-lawsone analogs were submitted to *in silico* screening using the program OSIRIS [18] to analyze their overall drug score and drug likeness potential as well as toxicity risks (mutagenic, irritant, tumorigenic and reproductive effects) [19] of the bis-lawsone analogs.

## 4. Conclusions

The present study reports the synthesis and leishmanicidal evaluation of a series of substituted bis-2-hydroxy-1,4-naphthoquinones prepared from lawsone. The *in vitro* cytotoxic activities of the derivatives synthesized were evaluated against *L. braziliensis*, where they showed a maximum effect greater than 60%. The bis-lawsone analogs **3a**, **3b**, **3e** and **3h** present efficacies and potencies similar to the reference drug pentamidine without cytotoxicity to the host cells. Finally, **3a** presented the activity 30 μmol/kg × 28 days (i.p.); the *L. amazonensis* lesion size on the infected ear of BALB/c mice decreased, but the number of parasites in the infected ear and draining lymph nodes did not decrease. In summary, these findings show that **3a**, **3e** and **3h** are antileishmanial drug candidates and suggest a useful starting point for rationally designing new agents against leishmaniasis. Clearly, further studies are necessary for exciting advances in the medicinal use of drug candidates in this class of secondary products.

## Supplementary Materials

Supplementary materials can be accessed at: http://www.mdpi.com/1420-3049/19/9/15180/s1.

## Supplementary Files

Supplementary File 1
