# Supplementary material for: Synthesis, Leishmanicidal Activity and Theoretical Evaluations of a Series of Substituted bis-2-Hydroxy-1,4-Naphthoquinones
Source: Molecules. 2014 Sep 22;19(9):15180–95. doi: 10.3390/molecules190915180 (PMC6271274; doi:10.3390/molecules190915180)

# Supplementary Materials

**Figure S1.** $^{1}$H (300 MHz) and $^{13}$C (75 MHz) NMR spectrum of (**3a**) in DMSO-$d_6$.

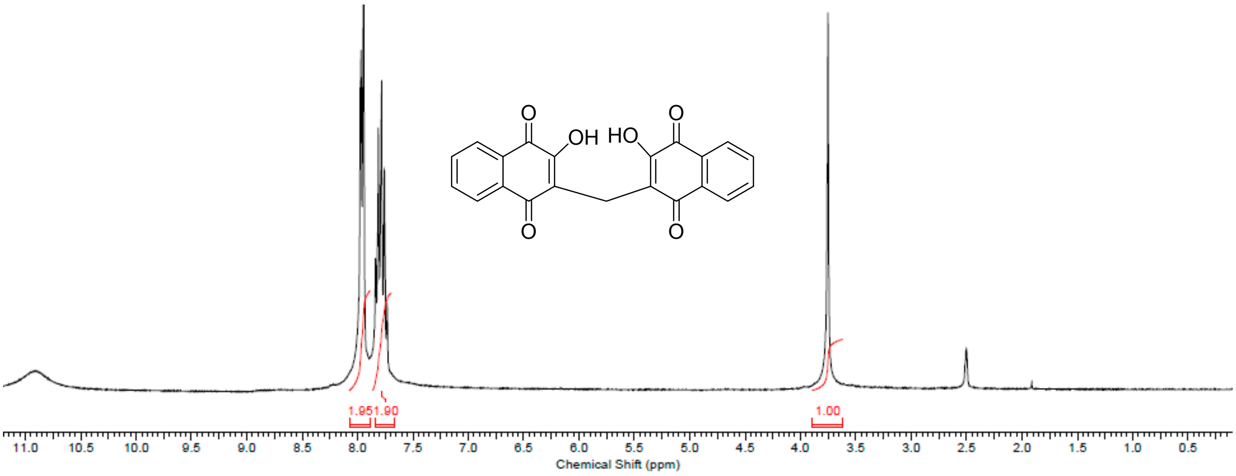


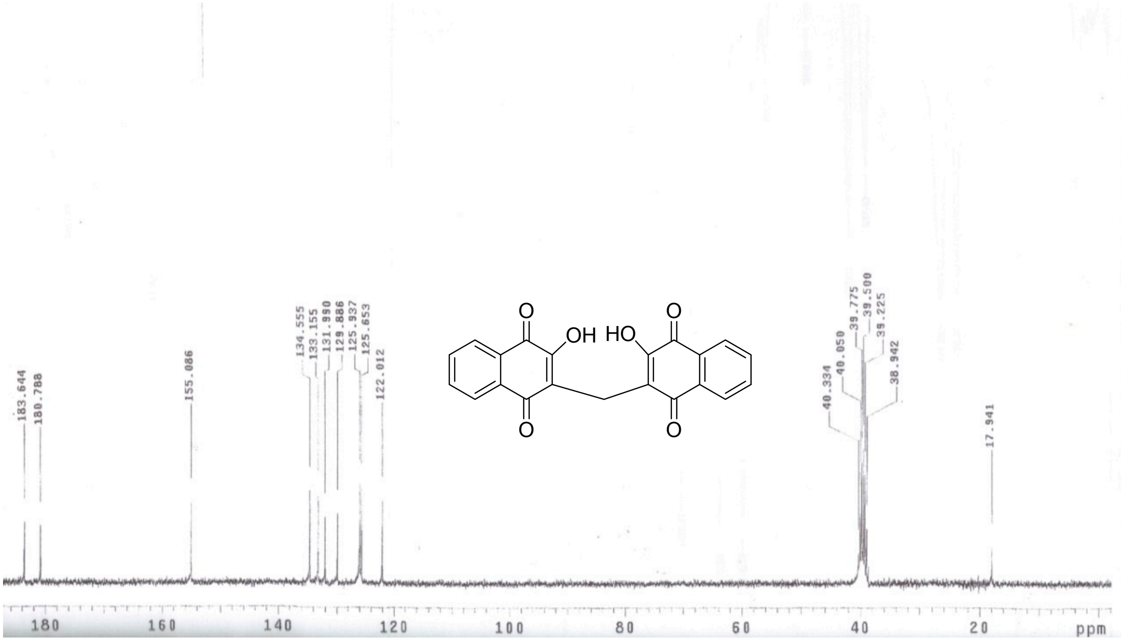

**Figure S2.** $^{1}H$ (300 MHz) and $^{13}C$ (75 MHz) NMR spectrum of (**3b**) in DMSO-$d_6$.

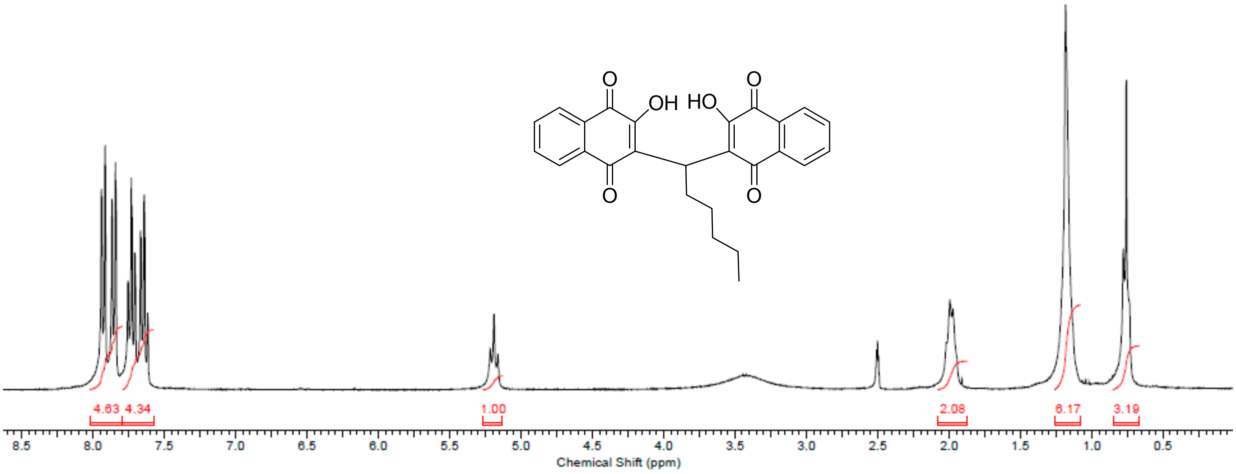


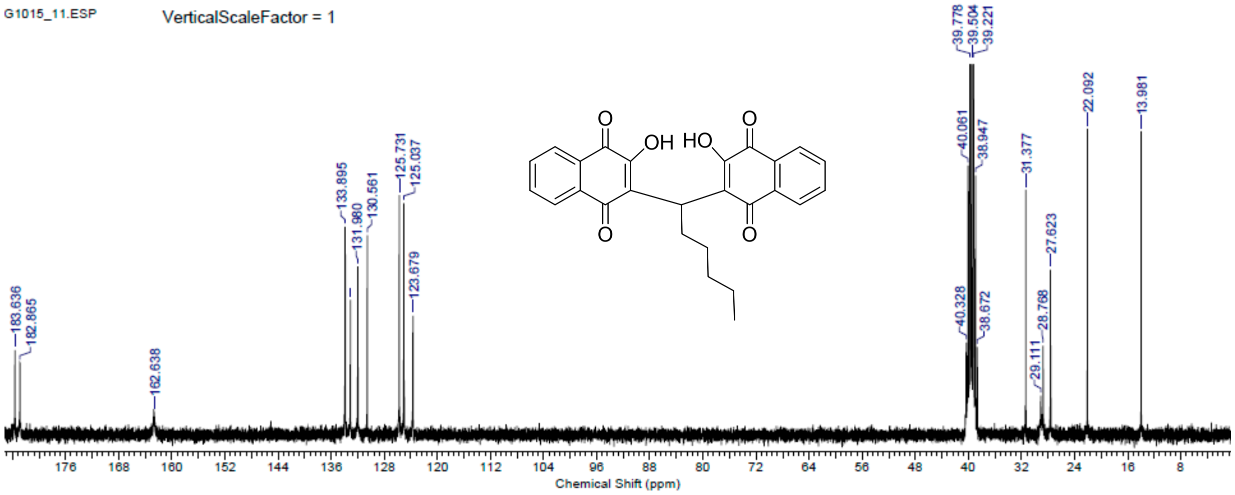

**Figure S3.** $^1$H (300 MHz) and $^{13}$C (75 MHz) NMR spectrum of (**3c**) in DMSO-$d_6$.

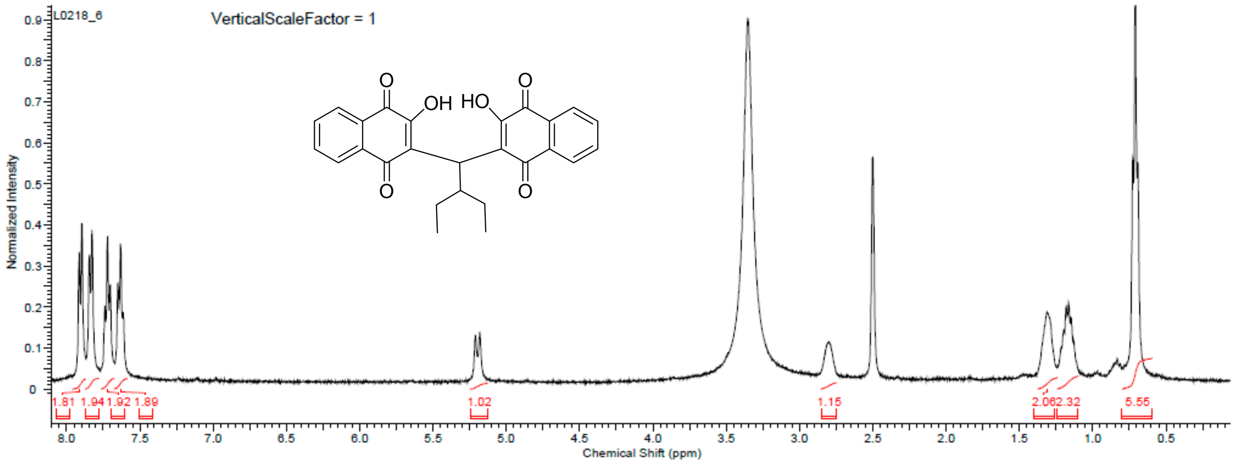


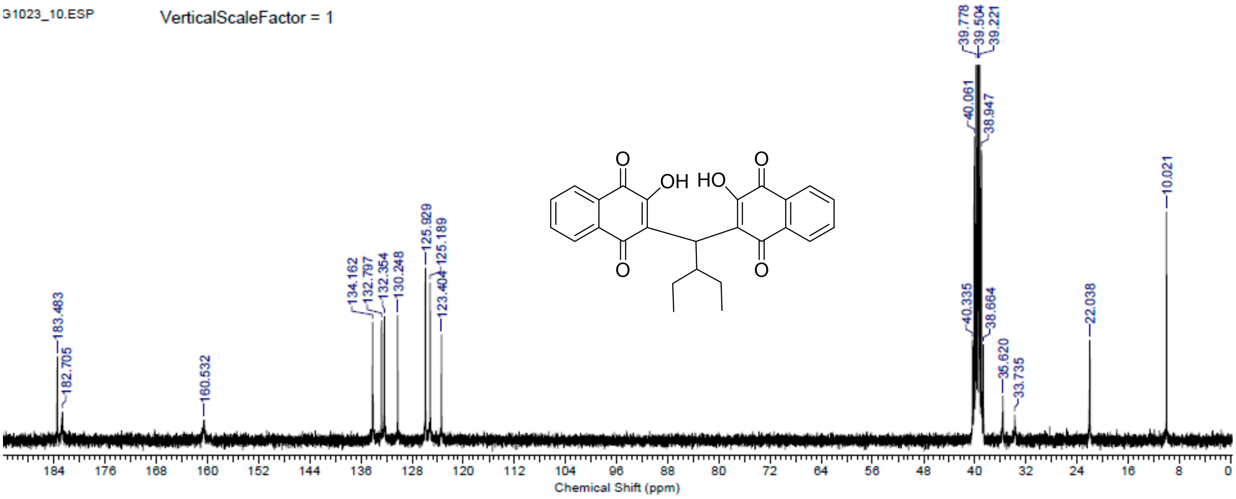

**Figure S4.** $^{1}$H (400 MHz) and $^{13}$C (100 MHz) NMR spectrum of (**3d**) in DMSO-$d_6$.

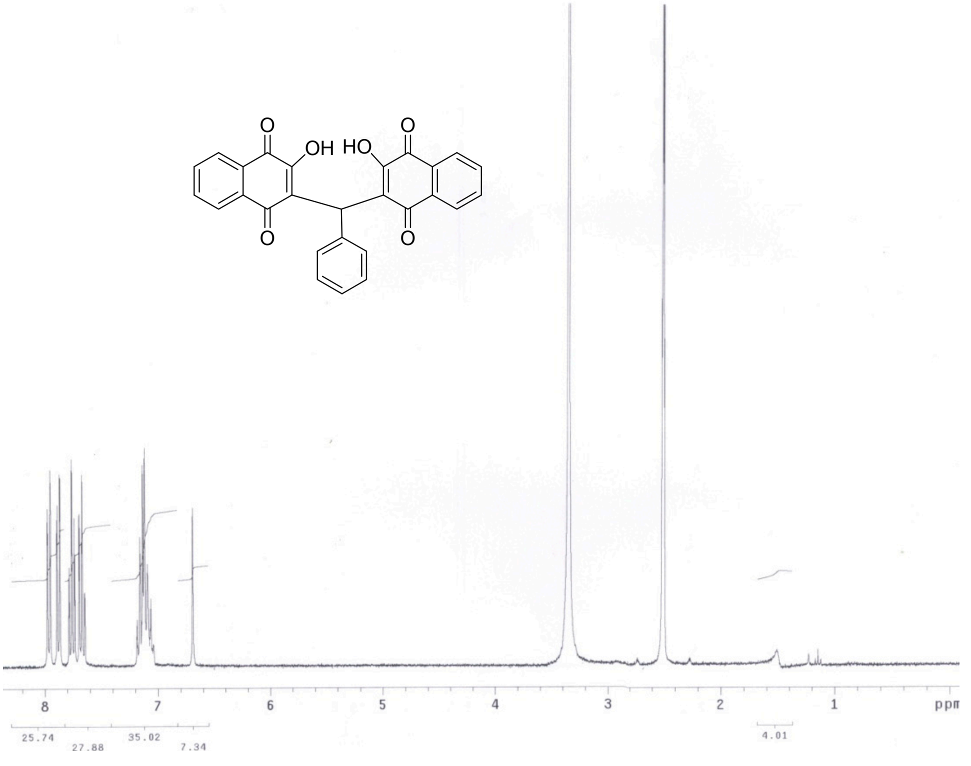


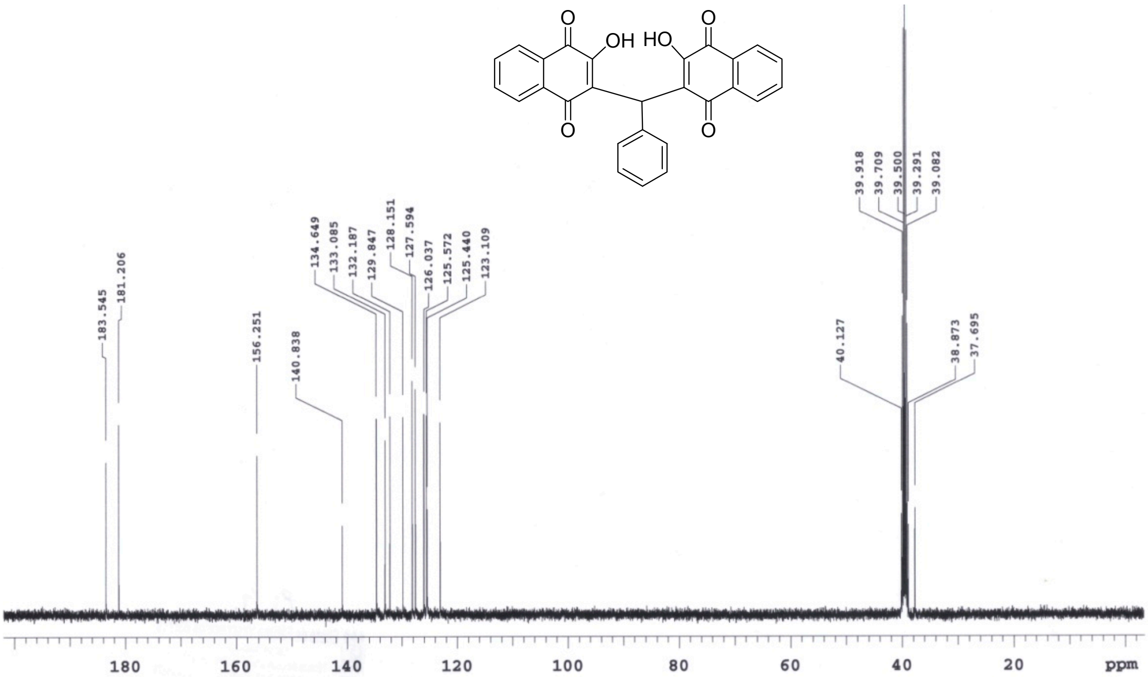

**Figure S5.** $^{1}$H (300 MHz) and $^{13}$C (75 MHz) NMR spectrum of (**3e**) in DMSO-$d_6$.

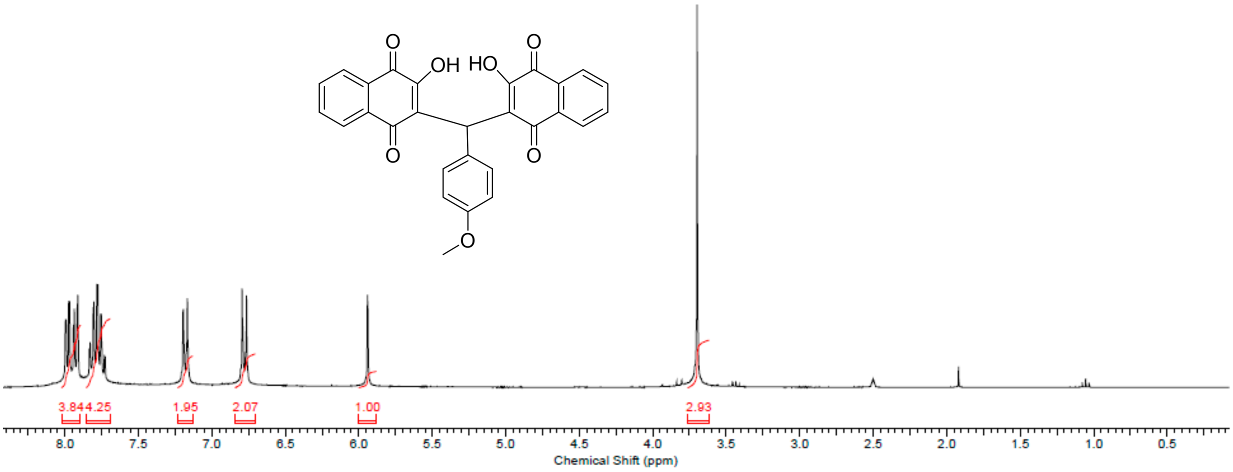


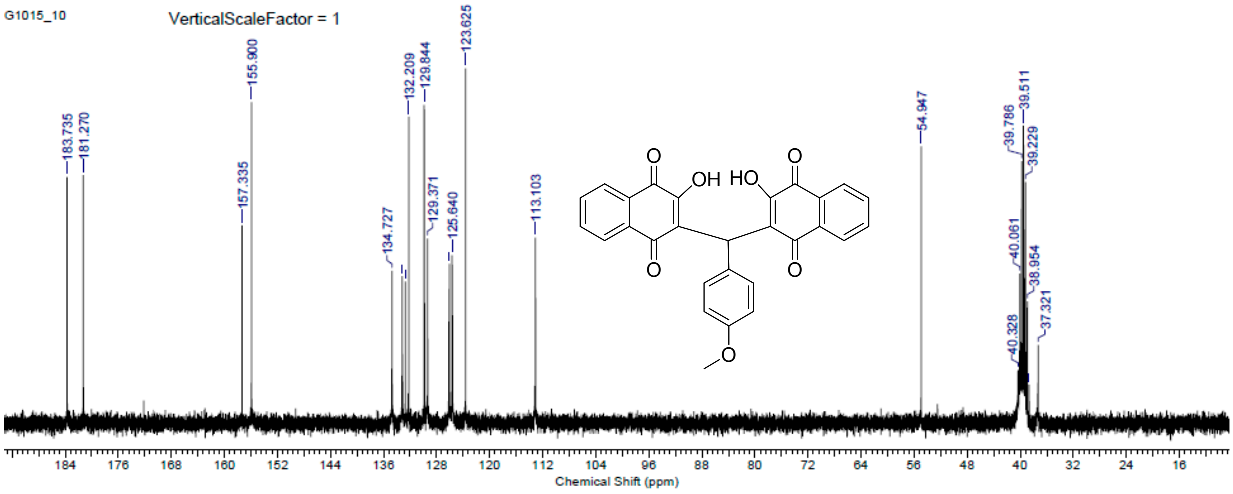

**Figure S6.** $^{1}$H (300 MHz) and $^{13}$C (75 MHz) NMR spectrum of (**3f**) in DMSO-$d_6$.

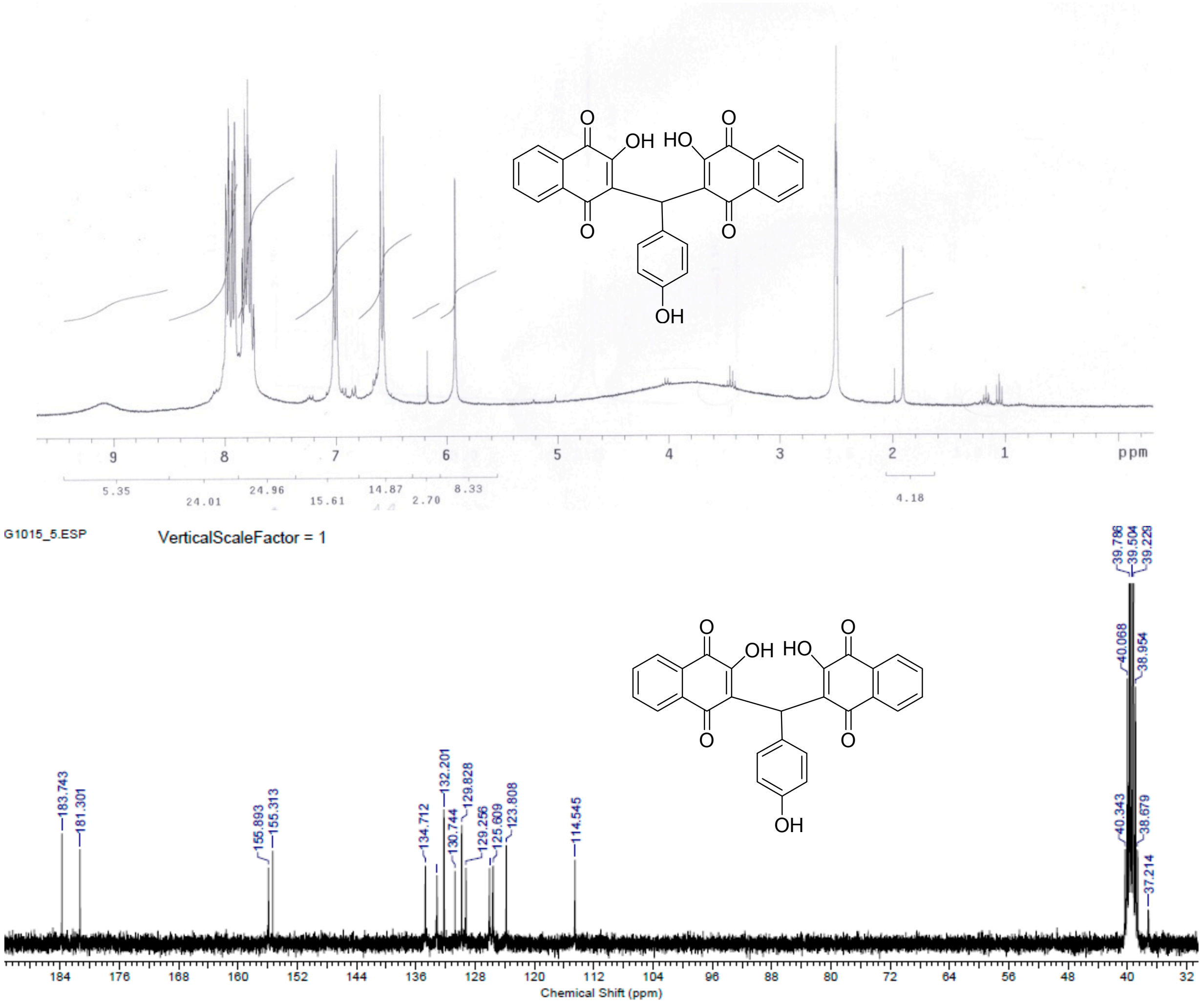

**Figure S7.** $^{1}H$ (300 MHz) and $^{13}C$ (75 MHz) NMR spectrum of (**3g**) in DMSO-$d_6$.

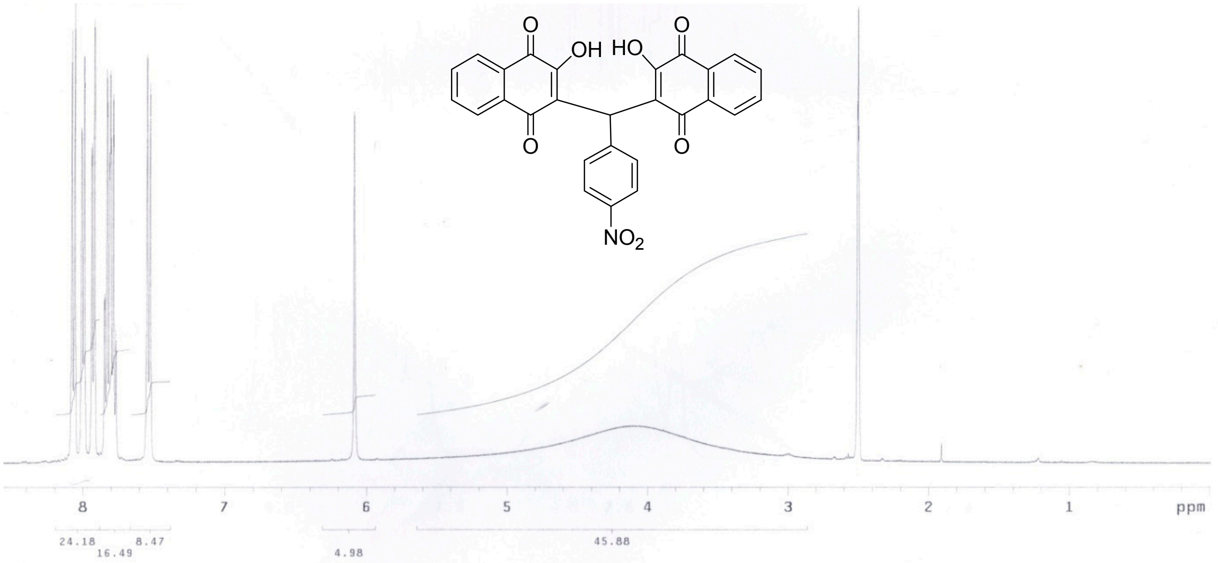


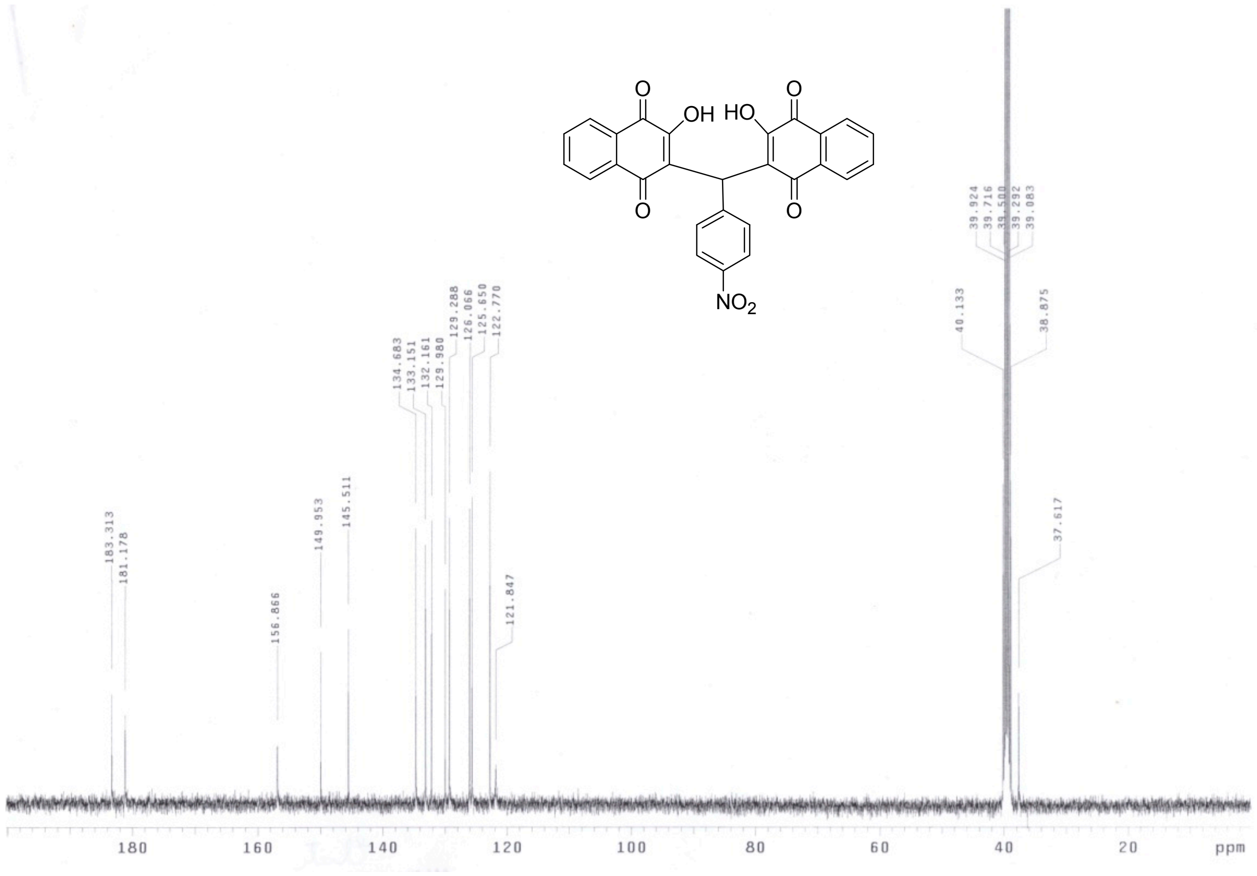

**Figure S8.** $^{1}H$ (400 MHz) and $^{13}C$ (100 MHz) NMR spectrum of (**3h**) in DMSO-$d_6$.

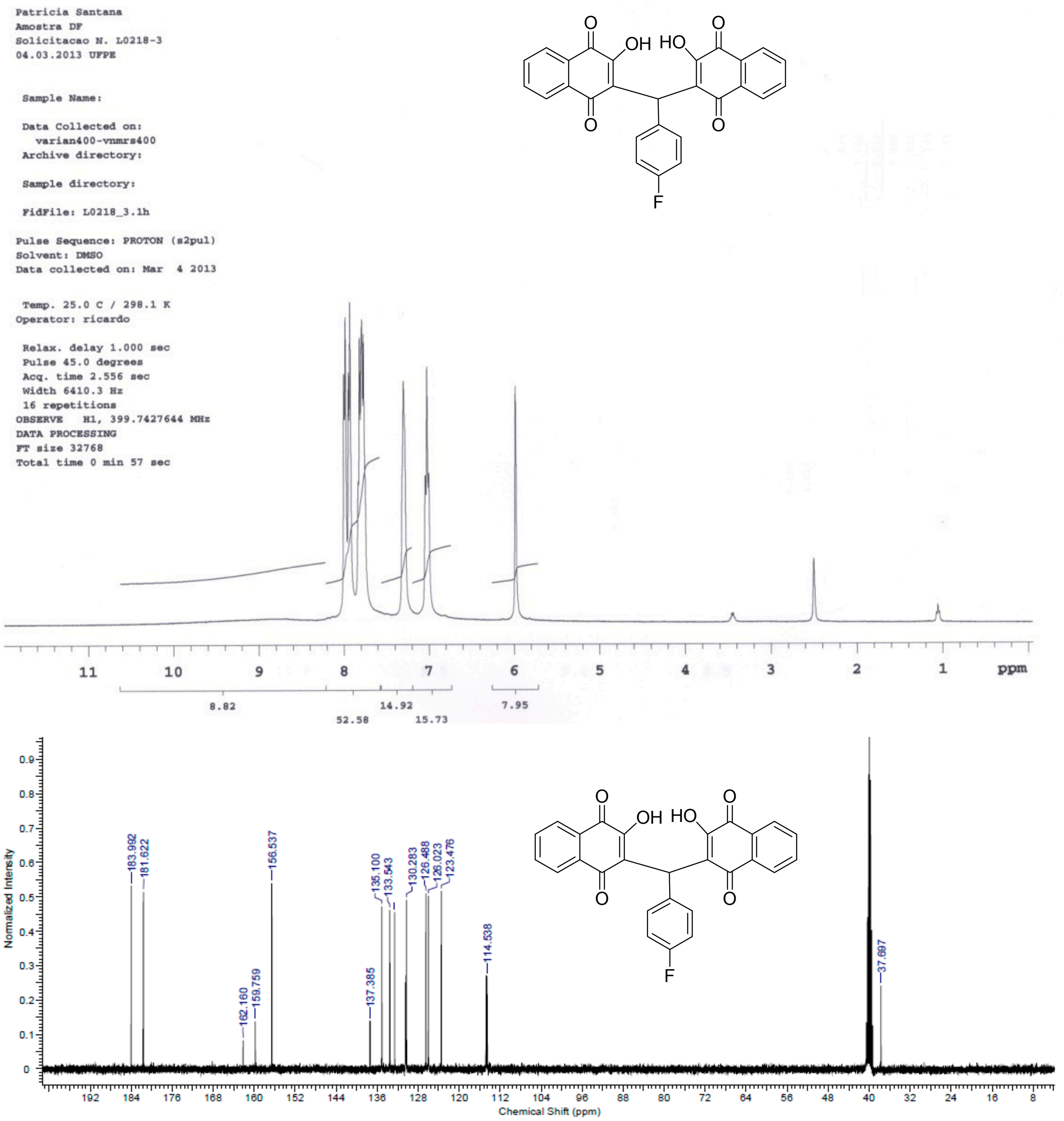

Supplement: Supplementary File 1 [file molecules-19-15180-s001.pdf]
